# Impact of Biological Therapies and Tofacitinib on Real-world Work Impairment in Inflammatory Bowel Disease Patients: A Prospective Study

**DOI:** 10.1093/ibd/izac002

**Published:** 2022-02-04

**Authors:** Pepijn W A Thomas, Nathan den Broeder, Monique Derikx, Wietske Kievit, Rachel L West, Maurice G V M Russel, Jeroen M Jansen, Tessa E H Römkens, Frank Hoentjen

**Affiliations:** Department of Gastroenterology and Hepatology, Radboud Institute for Molecular Life Sciences, Radboud University Medical Centre, Nijmegen, The Netherlands; Department of Gastroenterology and Hepatology, Radboud Institute for Molecular Life Sciences, Radboud University Medical Centre, Nijmegen, The Netherlands; Occupational Medicine, Department of Gastroenterology and Hepatology, Radboud University Medical Centre, Nijmegen, The Netherlands; Radboud Institute for Health Sciences, Department for Health Evidence, Radboud University Medical Centre, The Netherlands; Department of Gastroenterology and Hepatology, Franciscus Gasthuis and Vlietland, Rotterdam, The Netherlands; Department of Gastroenterology and Hepatology, Medical Spectrum Twente, Enschede, The Netherlands; Department of Gastroenterology and Hepatology, Onze Lieve Vrouwe Gasthuis, Amsterdam, The Netherlands; Department of Gastroenterology and Hepatology, Jeroen Bosch Hospital, ’s-Hertogenbosch, The Netherlands; Department of Gastroenterology and Hepatology, Radboud University Medical Centre, Nijmegen, The Netherlands and Division of Gastroenterology, Department of Medicine, University of Alberta, Edmonton, Canada

**Keywords:** work impairment, quality of life, patient-reported outcomes, inflammatory bowel disease, biologicals

## Abstract

**Background:**

There are limited real-world data on the change in total work impairment (TWI) in biological-treated patients with inflammatory bowel disease (IBD). This study aimed to evaluate the real-world effects of initiating biological therapy or tofacitinib on change in TWI in IBD patients.

**Methods:**

This multicenter prospective cohort study enrolled IBD patients who started treatment with biological therapy or tofacitinib. Subjects completed the work productivity and activity impairment (WPAI) questionnaire and short inflammatory bowel disease questionnaire at therapy initiation and at week 26. Total work impairment comprises working hours missed due to sick leave and impact of disease during working hours (range 0%-100%). Clinical disease activity was assessed using the Harvey-Bradshaw Index and Simple Clinical Colitis Activity Index (SCCAI).

**Results:**

We included 137 IBD patients for analyses (median age 38 years, 58% Crohn’s disease [CD]). The median baseline TWI was 50% and decreased by a median of 10%-points of points after 26 weeks. Patients with continued biological therapy or tofacitinib use, clinical disease activity at baseline, and clinical response or remission at week 26 showed a greater median TWI reduction (22%-points) than the remaining study patients (7%-points; *P* = .014). Ulcerative colitis (UC) and IBD-unclassified (IBD-U) patients showed a greater median TWI reduction (26%-points) than CD patients (6%-points); *P* = .041. Correlations were observed between decrease in TWI and decrease in SCCAI, decrease in fatigue and increase in quality of life.

**Conclusions:**

Work impairment in IBD patients decreased following biological therapy or tofacitinib initiation. Patients achieving clinical remission or response showed the greatest improvement, especially UC and IBD-U patients.

## Introduction

Inflammatory bowel disease (IBD) is a chronic and debilitating disease. It typically affects young adults and impairs well-being, quality of life, daily functioning, and work.^1^ Work disability rates among European patients with Crohn’s disease (CD) and ulcerative colitis (UC) range between 9% and 32%, compared with 7%-10% in control groups, reflecting the impact of IBD on work productivity.^2–6^ Average costs due to sick leave account for €2455 per patient per year and comprise 21% of the IBD population’s total costs per year, including both health care and work-related costs.^7^ Costs due to sick leave may equal €9.1 billion per year considering an estimated 3.7 million IBD patients in Europe.^8^

Biological therapies and tofacitinib are effective for inducing and maintaining remission in IBD patients. However, these therapies are expensive and are currently the main driver of the total IBD costs.^7^ Productivity losses due to sick leave are the second driver of costs in IBD patients. When including productivity losses due to impairment at work because of IBD, these costs may even be the main driver of the total IBD costs. A proportion of these costs may be reduced after initiating biological therapy or tofacitinib, leading to clinical improvement and resulting in reduced work impairment, especially in patients with a high disease burden.^9^

A few prospective studies in IBD patients have shown a reduction of total work impairment (TWI) and improved quality of life after biological therapy or tofacitinib initiation. The TWI decreased up to an average of 29%-points after 26 weeks,^10–13^ and these improvements were maintained for up to 6 years.^14^ Similarly, improvements were observed in quality of life.^10–14^ However, these results cannot be directly extrapolated to daily practice for several reasons. First, all of the described studies were phase 3 studies that included patients who are not representative of an average IBD population in daily practice, and studies were only tailored towards either CD or UC.^15^ In addition, change in TWI was only assessed for adalimumab, which raises the question whether similar outcomes can be expected for other biological therapies or tofacitinib. Moreover, work impairment is a multifactorial problem that may be caused by disease activity but also by other factors such as fatigue and social and work environment.^16^ Therefore, achieving adequate disease control may not always result in a reduction of TWI.^10–13^

This study aimed to evaluate the real-world effects of initiating biological therapy or tofacitinib on change in TWI in IBD patients. Secondly, we aimed to assess change in TWI in subgroups and asses correlations of change in TWI with disease activity, quality of life, and fatigue.

## Methods

### Study Design

This multicenter prospective cohort study assessed the change in TWI after introducing biological therapy or tofacitinib in IBD patients between May 1, 2019, and July 30, 2020.

### Study Population

Patients 18 years and older with an established diagnosis of CD, UC, or IBD-unclassified (IBD-U)^17^ were eligible if they initiated biological therapy (infliximab, adalimumab, golimumab, vedolizumab, or ustekinumab) or tofacitinib as recommended by their treating physician. Patients were prospectively enrolled after providing informed consent.

### Data Collection

We used the IBDREAM registry for data collection. IBDREAM systematically records prospective data from IBD patients in 1 academic and 4 nonacademic IBD care centers in the Netherlands, as described previously.^18–21^ At inclusion of this study, we recorded data on demographics, disease location and behavior according to the Montreal classification, prior IBD-related surgical procedures, previous and concomitant IBD medication use, clinical disease activity using the Harvey-Bradshaw Index (HBI) for CD and Simple Clinical Colitis Activity Index (SCCAI) for UC and IBD-U patients, C-reactive protein, fecal calprotectin, and endoscopic and radiological assessments if available. At baseline (biological initiation), during follow-up, and at 3 and 6 months postbiological initiation, we recorded clinical and biochemical disease activity, treatment changes, and IBD-related surgical procedures. Patients completed the following 2 questionnaires at the previously described time points: work productivity and activity impairment (WPAI) questionnaire and short IBD questionnaire (SIBDQ).

### Questionnaires

The WPAI questionnaire was used to evaluate (1) work time missed due to IBD (absenteeism); (2) impairment at work due to IBD (presenteeism); (3) TWI due to IBD, which is calculated as follows: (absenteeism + ((1 − absenteeism) × presenteeism)); and (4) total activity impairment. Unemployed patients only answered questions relating to employment status and daily activities. The WPAI domain scores range from 0%-100%, with higher scores indicating greater impairment in work and activities.^22^ Change in WPAI domain scores ranges from −100%-points to 100%-points. A reduction of 7%-points in a WPAI domain score was considered the minimal clinical important difference (MCID).^23^

The SIBDQ is a validated 10-item instrument that measures quality of life in patients with IBD. Each question consists of a Likert scale ranging from 1-7. Scores range from 10-70, with higher scores implying better quality of life.^24^ A 9-point improvement was considered the MCID.^10^ Within the SIBDQ, 1 question focusses on fatigue in which patients can rank fatigue from 1 (“all the time”) to 7 (“none of the time”.) Patients scoring ≤4 are classified as fatigued.^25^

### Outcomes and Definitions

The primary outcome of this study was the change in TWI between baseline and week 26. Secondary outcomes included change in separate domains of the WPAI questionnaire, the proportion of patients with a paid job at baseline, the proportion of patients achieving MCID for TWI and total SIBDQ score, quality of life measured by SIBDQ, fatigue measured in the SIBDQ, clinical remission, clinical response rates, subgroup analyses for change in TWI for UC/IBD-U vs CD and for patients with clinical benefit of continued biological therapy use vs the remaining study patients and TWI at baseline, week 13, and week 26 for patients included pre-COVID-19 vs during COVID-19. Clinical benefit of continued biological therapy or tofacitinib use was defined as patients with clinical disease activity at baseline and clinical response or remission at week 26 who were still on continued use of biological therapy or tofacitinib at week 26. Clinical remission was defined as an HBI score <5 for CD patients or an SCCAI score <3 for UC and IBD-U patients. Clinical response was defined as a reduction of ≥3 points in HBI compared with baseline or ≥2 points in SCCAI compared with baseline. Biochemical disease activity was defined as fecal calprotectin >200 µg/g and/or C-reactive protein >10 mg/L. Patients with missing values for biochemical disease measures at week 13 and week 26 were considered nonresponders. Objectively confirmed disease activity was defined as C-reactive protein >10 mg/L, fecal calprotectin >200 µg/g, and/or transmural inflammation on magnetic resonance imaging or computed tomography and/or presence of ulcers during endoscopic assessment. We used the date of lockdown following the first wave in the Netherlands (March 12, 2020) to stratify patients into 2 groups: (1) the pre-COVID-19 group was defined as patients included before March 12, 2020, and (2) the COVID-19 group was defined as patients included from March 12, 2020, and onward. As of May 11, 2020, restrictions due to COVID-19 were slowly reduced in a stepwise manner.

### Statistical Analysis

Participants were considered dropouts if they discontinued biological therapy or tofacitinib during the induction phase and did not start a subsequent biological or tofacitinib. Analyses were performed for participants who were employed at some time during the study period. Normally distributed values were presented as mean with standard deviation and compared using the Student *t* test. Non-normally distributed variables were presented as median with interquartile range (IQR) and compared using the Mann-Whitney *U* test. Categorical variables were presented as percentages and compared using χ^2^ , Fisher exact test, or McNemar test. Regression on ranks or signed-ranks was used to estimate the pooled effects of the multiple imputed data sets for independent samples and paired samples for each WPAI domain and SIBDQ total score, respectively. Subgroup analyses for change in TWI over time were performed and included continued clinical benefit of biological therapy use vs the remaining study participants, CD vs UC and IBD-U patients, participants included pre-COVID-19 vs during COVID-19 and patients receiving intravenously administered therapies vs self-administered therapies. Spearman rank correlation was used to measure the correlation between change in TWI and change in disease activity, change in quality of life, change in fatigue score, and age at baseline. Missing data for clinical disease activity, absenteeism, presenteeism, total activity impairment, fatigue score in the SIBDQ, and the total SIBDQ score were imputed by performing multiple imputations by chained equations using predictive mean matching. We used 50 iterations per variable and constructed 10 imputed data sets. Missing data were assumed to be missing at random. The percentage of missing data per variable ranged from 2% to 16%. The following variables were included in the imputation model: IBD subtype, participating center, gender, smoking status, body mass index, HBI scores, SCCAI scores, C-reactive protein assessments, WPAI domains, SIBDQ total score, and SIBDQ fatigue score. Two-sided *P* values <0.05 were considered statistically significant. The SPSS Statistics (IBM, version 25.0) was used for statistical analyses.

### Ethical Considerations

This study was reviewed and approved by the Radboudumc Committee on Research Involving Human Subjects (ref. 2018-4110).

## Results

### Study Population

#### Inclusion and response rate

In total, 194 consecutive IBD patients were enrolled. Data from 3 patients were not used in the analysis because they discontinued biological therapy during the induction phase and did not start a subsequent biological or tofacitinib. Overall, 17 patients (9%) reported full work disability, and 7 patients (4%) partial work disability. At baseline, 128 patients (67%) had a paid job, of whom 4 patients (3%) experienced partial work disability. During follow-up, 7 patients lost their job, and 9 patients started employment. In total, 137 patients were included for further analyses. Response rates to questionnaires at baseline, week 13, and week 26 were 97%, 86%, and 89%, respectively. Of these patients, 108 (79%) completed questionnaires at each consecutive time point.

#### Baseline characteristics

In our cohort, the median age was 38 years (IQR, 27-48), 58% had CD, and 50% were male. At baseline, 43 patients (31%) were started on adalimumab, 42 patients (31%) on infliximab, 25 patients (18%) on ustekinumab, 23 patients (17%) on vedolizumab, 3 patients (2%) on tofacitinib, and 1 patient (1%) on golimumab. Nearly 50% of patients were naïve to biologicals. Corticosteroids were used in 31% of patients, 13% used budesonide, and 49% used a concomitant immunomodulator. In CD patients, 30% had stricturing disease, and 28% had penetrating disease (Table 1).

**Table 1.** Baseline characteristics.

		Study Population
		N = 137
Sex, male	N (%)	68 (49.6)
Age, in years	Median (IQR)	38.1 (26.6–48.2)
Body Mass Index, kg/m^2^	Mean ± SD	23.2 ± 4.5
Employed	N (%)	128 (93.4)
Disease duration, in years	Median (IQR)	7.0 (2.0–15.0)
IBD diagnosis		
Crohn’s disease	N (%)	80 (58.4)
Ulcerative Colitis	N (%)	52 (38.0)
IBD-U	N (%)	5 (3.6)
Montreal Classification Crohn’s disease		
Disease location		
Ileum	N (%)	23 (28.8)
Colon	N (%)	14 (10.2)
Ileocolonic	N (%)	42 (52.5)
Upper Gastrointestinal tract Involvement	N (%)	16 (20.0)
Disease behavior		
Stricturing	N (%)	24 (30.0)
Penetrating	N (%)	22 (27.5)
Perianal involvement	N (%)	15 (18.8)
Disease extent ulcerative colitis/ IBD-U		
Proctitis	N (%)	1 (1.8)
Left-sided	N (%)	22 (38.6)
Pancolitis	N (%)	34 (59.6)
Current Biological		
Adalimumab	N (%)	43 (31.4)
Infliximab	N (%)	42 (30.7)
Ustekinumab	N (%)	25 (18.2)
Vedolizumab	N (%)	23 (16.8)
Tofacitinib	N (%)	3 (2.2)
Golimumab	N (%)	1 (0.7)
Prior biological		
None	N (%)	67 (48.9)
Anti-TNF		
≥1	N (%)	67 (48.9)
≥2	N (%)	19 (13.9)
Vedolizumab	N (%)	13 (9.5)
Ustekinumab	N (%)	6 (4.4)
Prior small molecule therapy		
Tofacitinib	N (%)	3 (2.2)
Concomitant medication		
Mesalamine	N (%)	43 (31.4)
Budesonide	N (%)	18 (13.1)
Corticosteroids	N (%)	43 (31.4)
Immunomodulator	N (%)	67 (48.9)
At least one prior intestinal resection	N (%)	18 (13.1)
Ileostomy/Colostomy	N (%)	5 (3.6)
Smoking status		
Active smoker	N (%)	22 (16.1)
Previous smoker	N (%)	35 (25.5)
Never smoked	N (%)	77 (56.2)
Missing	N (%)	3 (2.2)
Education level		
< Secondary school graduate	N (%)	4 (2.9)
Secondary school graduate	N (%)	67 (48.9)
Post-secondary school degree	N (%)	66 (48.2)
Disease activity		
Simple Clinical Colitis Activity Index	Median (IQR)	6.0 (3.0–8.0)
Harvey-Bradshaw Index	Median (IQR)	4.0 (2.0–6.2)
Clinically active disease activity^a^	N (%)	82 (59.9)
Objectively confirmed disease activity^b^	N (%)	120 (87.6)

Clinically active disease activity was defined as a Harvey-Bradshaw Index >4 for Crohn’s disease or Simple Clinical Colitis Activity Index >2 for ulcerative colitis or inflammatory bowel disease-unclassified

Objectively confirmed disease activity was defined as fecal calprotectin >200 µg/g and/or transmural inflammation on magnetic resonance imaging or computed tomography and/or presence of ulcers during endoscopic assessment.

Abbreviations: anti-TNF, anti-tumour necrosis factor; IBD-U, inflammatory bowel disease-unclassified

### Clinical Outcomes

At baseline, 82 patients (59.9%) had clinically active disease based on HBI and SCCAI scores, 84 patients (61%) had biochemically active disease, and 131 patients (96%) had disease activity based on either HBI or SCCAI scores and/or based on fecal calprotectin, endoscopy, or radiology. Five patients showed clinical symptoms requiring biological therapy or tofacitinib but did not achieve HBI >5 or SCCAI >2. One patient restarted adalimumab after developing adverse events to mercaptopurine. Clinical remission rates at week 13 and week 26 were 72% (*n* = 99) and 78% (*n *= 107), respectively. Clinical response rates at week 13 and week 26 were 53% (*n* = 72) and 53% (*n* = 73), respectively. In patients with biochemical disease activity at baseline (*n* = 82), biochemical remission rates at week 13 and week 26 were 57% (*n* = 48 of 84) and 52% (*n* = 44 of 84), respectively. During follow-up, 22 patients (16%) discontinued biological therapy or tofacitinib after a median 14 weeks (IQR, 10-21). Reasons for discontinuation included insufficient effect (*n* = 20) and adverse events (*n *= 2). Fifteen patients started a subsequent biological or tofacitinib during follow-up. One patient also discontinued a second biological during the study due to an adverse event. In total, 7 patients (5%) required intestinal surgery.

### Work Productivity and Activity Impairment

#### Change in total work impairment

Baseline TWI was 50.0% (IQR, 20.0-89.5) and decreased by a median of 10.0%-points (IQR, −9.5-46.7; *P* < .001) after 26 weeks. Furthermore, 77 patients (56%) reported a clinically relevant decrease in TWI of ≥7%-points at week 26. In patients in clinical remission at week 26 (*n* = 107), the median TWI was 20.1% (IQR, 0.0-58.2).

#### Clinically effective continued use of biological therapy or tofacitinib

**Figure 1.**
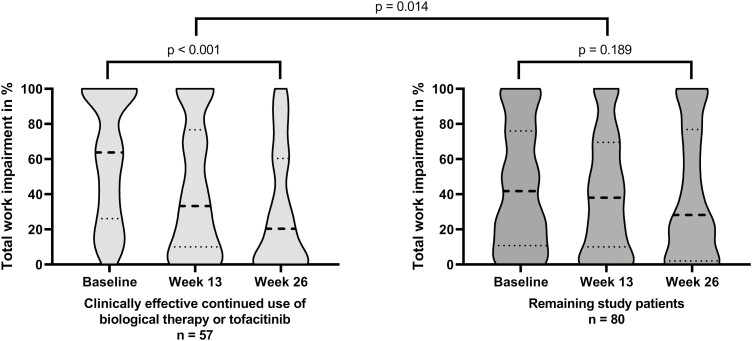
Violin plots for total work impairment stratified for patients with clinical disease activity at baseline (Harvey-Bradshaw Index (HBI) ≥5 or Simple Clinical Colitis Activity Index (SCCAI) ≥3) and clinical response (HBI reduction ≥3 or SCCAI reduction ≥2) or clinical remission (HBI < 5 or SCCAI < 3) at week 26, and continued use of the of biological therapy or tofacitinib at week 26 (n = 57) compared with the remaining patients in the study population (n = 80). The thick dotted line represents the median value and the thin dotted lines represent the 25^th^ and 75^th^ percentiles.

Patients with clinical disease activity at baseline and clinical response or remission at week 26 who were still on continued use of biological therapy or tofacitinib at week 26 (*n* = 57) showed a median TWI decrease of 22.2%-points (IQR, −1.5-68.0; *P* < .001) compared with 6.8%-points (IQR, −17.0-36.7; *P* = .189) in the remaining study participants (*n* = 80; *P* = .014; Fig. 1). A clinically relevant decrease in TWI was reported in 65% (*n* = 37) of patients on continued clinically effective biological therapy or tofacitinib compared with 50% (*n *= 40) in the remaining study participants (*P* = .083).

#### CD patients vs UC/IBD-U patients

**Figure 2.**
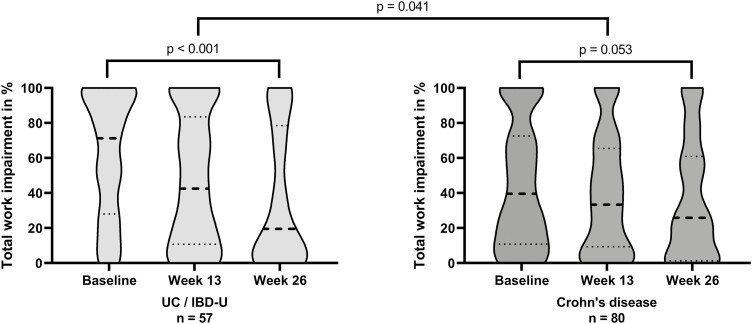
Violin plots for total work impairment stratified for patients diagnosed with ulcerative colitis and inflammatory bowel disease-unclassified (*n* = 57) vs Crohn’s disease (*n *= 80). The thick dotted line represents the median value, and the thin dotted lines represent the 25th and 75th percentiles. Abbreviations: UC, ulcerative colitis; IBD-U, inflammatory bowel disease-unclassified.

UC and IBD-U patients (*n* = 57) showed a median TWI decrease of 25.6%-points (IQR, −4.8-61.7; *P* < .001) compared with only 5.9%-points (IQR, −11.7-34.0; *P* = .053) in CD patients (*n* = 80) (*P* = .041; Fig. 2). A clinically relevant decrease in TWI was reported in 67% (*n *= 38) of UC/IBD-U patients compared with 49% (*n* = 39) in CD patients (*P* = .037).

#### Change in other WPAI domains

After 26 weeks, presenteeism decreased by a median of 9.0%-points (IQR, −10.0-31.3; *P* = .010), and daily activity impairment decreased by a median of 17.0%-points (IQR, −1.0-40.5; *P* < .001). Absenteeism decreased statistically significantly (median 0.0%-points (IQR, 0.0-29.2; *P* = .006)), but the median change was 0.0%-points, as most patients did not report absenteeism at baseline. In patients that reported absenteeism at baseline (*n* = 68; 49.6%), we observed a median decrease of 28.6%-points in absenteeism (IQR, 8.4–94.7; *P* < .001) at week 26. The three-monthly scores for each WPAI domain are presented in Table 2. We assessed factors that potentially impacted our findings. First, when comparing the median TWI at baseline, week 13, and week 26 for patients included pre-COVID-19 (*n* = 104) and during COVID-19 (*n* = 33), we did not find any statistically significant differences (Supplementary Table 1). Second, when we compared therapies with intravenous administration vs self-administered medication, we did not find statistically significant differences in absenteeism (data not shown).

**Table 2.** Work Productivity and Activity Impairment Questionnaire domain scores at biological initiation and after 13 and 26 weeks.

		Baseline	Week 13	Week 26	Reduction between week 0-26	*P*
Absenteeism, in %	Median (IQR)	1.2 (0.0–60.4)	0.0 (0.0–33.8)	0.0 (0.0–20.0)	0.0 (0.0–29.2)	0.006
Presenteeism, in %	Median (IQR)	30.0 (10.0–60.0)	20.0 (4.0–40.5)	20.0 (0.0–47.0)	9.0 (-10–31.3)	0.010
Total work impairment, in %	Median (IQR)	50.0 (20.0–89.5)	36.6 (10.0–70.0)	22.3 (0.0–65.5)	10.0 (-9.5–46.7)	<0.001
Total activity impairment, in %	Median (IQR)	50.0 (30.0–70.0)	30.0 (10.0–53.5)	25.0 (9.5–59.5)	17.0 (-1.0–40.5)	<0.001

Absenteeism and total work impairment was available in 137 patients. Total activity impairment was available in 137 patients. Presenteeism was available at baseline in 109 patients, at week 13 in 118 patients, and at week 26 in 126 patients.

### Quality of Life

During follow-up, SIBDQ total scores increased by a median of 7.4 (IQR, 0.1-15.4; *P* < .001). Similar changes in SIBDQ total score per subgroup were seen compared with changes in TWI (Supplementary Figures 1-3). Overall, predefined clinically relevant improvement of ≥9 points was observed in 55 patients (40%). Insufficient improvement in quality of life was seen in 45 patients (33%), decrease in quality of life in 25 patients (18%), and 12 patients (9%) showed no change. Fewer patients reported fatigue at week 26 (*n* = 73; 53%) compared with baseline (*n* = 97; 71%; *P* < .001).

### Correlational Analyses Total Work Impairment

We found weak correlations between decrease in TWI, decrease in SCCAI (r = 0.350; *P* = .010), and decrease in fatigue (r = 0.275; *P* = .005); we found a moderate correlation between decrease in TWI and increase in SIBDQ total scores (r = -0.425; *P* < .001; Supplementary Figs. 3-5). We found no correlation with change in TWI and age at baseline or change in HBI.

## Discussion

This is the first multicenter, prospective, real-world cohort study that evaluated changes in work impairment in biological- or tofacitinib-initiated IBD patients. Overall, TWI decreased by a median of 10%-points after 26 weeks of treatment. Especially IBD patients with clinical disease activity at baseline and subsequent clinical improvement on biological therapy or tofacitinib had a clear benefit. Patients with UC and IBD-U showed a greater median decrease in TWI compared with CD patients. Lastly, we observed correlations between change in TWI and SCCAI scores, fatigue scores, and SIBDQ total scores.

Reducing work impairment and improving quality of life are important treatment goals in IBD patients. Low quality of life can be predictive for permanent work disability in CD patients.^26^ An improvement in both work impairment and quality of life was observed 26 weeks after introducing biological therapy or tofacitinib. Patients with clinically active disease at baseline who showed clinical response or remission after 26 weeks of biological therapy or tofacitinib reported a greater reduction of work impairment than the remaining study participants. This is in line with other studies that confirmed that disease burden correlates with work impairment.^10–13^ Prior studies even showed a clinically relevant improvement in work impairment as early as 4 weeks after adalimumab initiation.^10,11^ Combined, these findings underline that adequate disease control may result in improvement of work impairment.^10–13,27^ The median TWI of 50% at baseline indicates the significant disease burden on the ability to work in biological- or tofacitinib-initiated IBD patients. For UC patients in our cohort, work impairment (median 71%, mean 61%) was similar to adalimumab-initiated UC patients (mean 59%).^12^ Work impairment in biological- or tofacitinib-initiated UC patients is higher compared with UC patients initiated on mesalamine (42%), likely reflecting the severity and impact of disease in patients requiring biological therapy or tofacitinib.^27^

Work impairment is a complex problem that may be caused by the underlying IBD but also by mental health and work-related factors. Indeed, at the end of follow-up in our study, patients in clinical remission still reported a median TWI of 20%, suggesting that a proportion of work impairment may not be related to disease activity. Previous studies showed that fatigue is often the main reason for work impairment in IBD patients and is difficult to treat, considering it is still frequently present in IBD patients with quiescent disease.^9,28–30^ Our finding that fatigue and quality of life were correlated with work impairment is in line with a recent study that showed that work productivity loss was strongly determined by fatigue and decreased quality of life.^9^

We observed greater reductions in work impairment in UC and IBD-U patients compared with CD patients. These changes in UC patients are consistent with a previous phase 3 study including adalimumab-treated UC patients that reported an average reduction of 29%-points after 26 weeks.^12^ However, the reduction in TWI in CD patients in our study was significantly lower than reported in previous studies.^10,11,13,14^ Methodologic differences may explain this discrepancy. Total work impairment at baseline in our cohort of CD patients was lower (median 42%) compared with previous studies reporting an average TWI ranging from 48% to 70%. Previous studies that assessed the effect of biological therapy on work impairment included a strongly selected population with active clinical disease. We consecutively included all IBD patients initiated on biological therapy or tofacitinib, independent of the degree of disease activity, which more accurately reflects a real-world setting and identifies the burden of work impairment in both patients with clinically active disease and patients with limited clinical symptoms. We did find a correlation between change in clinical disease activity and work impairment in UC patients—but not in CD patients. This may be explained by the poor correlation between symptoms and disease activity in CD patients.^31,32^

Inflammatory bowel disease patients frequently experience work impairment.^2^ Work impairment includes sick leave but more often concerns reduced work productivity on the work floor due to IBD.^33^ Health care providers should be more aware of reduced work productivity and identify disabling factors that contribute to work impairment. Therefore, rapid induction of remission and the use of biological therapy or tofacitinib with the highest likelihood of success is important. Although this decision seems straightforward, physicians may be restricted by regulations and patients’ preferences. Patients may additionally require tailor-made guidance to further reduce work impairment, including physiotherapy and guidance on coping with the underlying disease and fatigue.

Strengths of this multicenter study include the prospective design and high response rate (≥86%) per questionnaire. This is the first study that reports changes in work impairment in consecutively enrolled biological-initiated IBD patients, providing more insight into the burden of disease in a general IBD population initiated on biological therapy or tofacitinib. The real-world setting allows for the extrapolation of outcomes to daily IBD practice. Lastly, we included all currently registered biologicals and tofacitinib, whereas previous studies only assessed the change in work impairment after adalimumab initiation. Limitations include the WPAI questionnaire that only includes work impairment in the preceding week instead of providing cumulative work impairment over a more extended recall period. This may have resulted in some imprecision, especially considering the relapsing and remitting disease course of IBD. However, other questionnaires such as the Productivity Cost Questionnaire can also increase the risk of recall bias.^34^ A 2-week recall period has been reported to minimize under-reporting of health-related productivity losses.^35^ Finally, no control group was included, and therefore no causal relationship on the effect of adequate disease control through the use of biological therapies or tofacitinib can be established. However, we found a greater reduction in TWI in patients with clinical benefit from biological therapy or tofacitinib use, which suggests adequate disease control does result in less work impairment. In addition, it was not ethical or possible to construct a control group of patients with active inflammation and withholding the required treatment.

## Conclusions

In conclusion, work impairment in IBD patients decreased following biological therapy or tofacitinib initiation. Patients with evident clinical benefit during ongoing use of biological therapy or tofacitinib showed the greatest improvement, especially in UC and IBD-U patients. These results underline the importance of disease control to improve work productivity and participation while additional tailored management may be required to further reduce work impairment.

## Supplementary Material

izac002_suppl_Supplementary_Material
