# Supplementary material for: Impact of Biological Therapies and Tofacitinib on Real-world Work Impairment in Inflammatory Bowel Disease Patients: A Prospective Study
Source: Inflamm Bowel Dis. 2022 Feb 4;28(12):1813–20. doi: 10.1093/ibd/izac002 (PMC9713499; doi:10.1093/ibd/izac002)
Supplement: izac002_suppl_Supplementary_Material [file izac002_suppl_supplementary_material.docx]

**Supplementary Files**

| **Total work impairment in %** |  | **Pre-COVID-19 (n=104)** | **COVID-19 (n=33)** | **p-value** |
| --- | --- | --- | --- | --- |
| **Baseline** | **Median (IQR)** | 50.1 (19.4 - 93.5) | 51.0 (19.9 - 82.2) | 0.849 |
| **Week 13** | **Median (IQR)** | 38.4 (10.0 72.9) | 30.2 (8.5 - 50.5) | 0.266 |
| **Week 26** | **Median (IQR)** | 24.4 (5.2 - 68.1) | 17.5 (0.0 - 53.9) | 0.213 |
| **Supplementary Table 1.** Total work impairment in patients included pre-COVID-19 and during COVID-19 in the Netherlands. 71 out of 104 patients that were included pre-COVID-19 had their week 26 assessment during the COVID-19 pandemic. IQR = interquartile range | | | | |


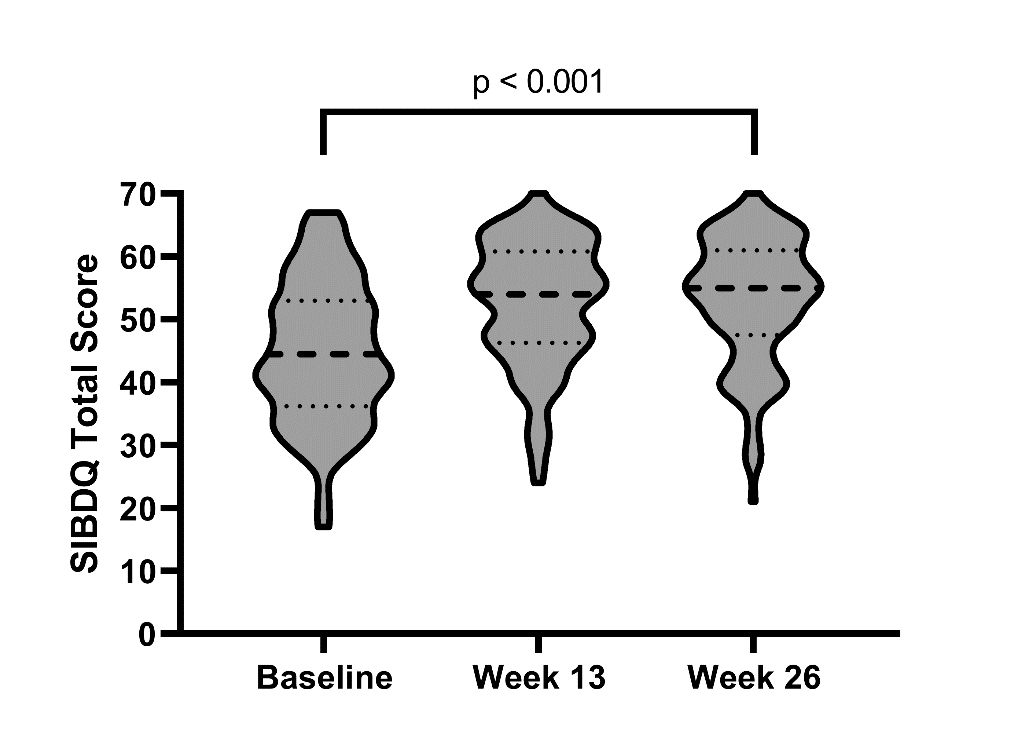


**Supplementary Figure 1.** Total short inflammatory bowel disease questionnaire (SIBDQ) scores at baseline, week 13 and week 26. The thick dotted line represents the median value and the thin dotted lines represent the 25th and 75th percentiles.

|  |  | Baseline | Week 13 | Week 26 | Improvement between week 0 - 26 | p-value |
| --- | --- | --- | --- | --- | --- | --- |
| SIBDQ Total Score | Median (IQR) | 44.5 (36.4 – 53.0) | 54.0 (46.6 – 60.6) | 55.0 (48.0 – 61.0) | 7.4 (0.1 – 15.4) | <0.001 |
| Supplementary Table 1. Short Inflammatory Bowel Disease Questionnaires total scores. SIBDQ = short inflammatory bowel disease questionnaire. | | | | | | |


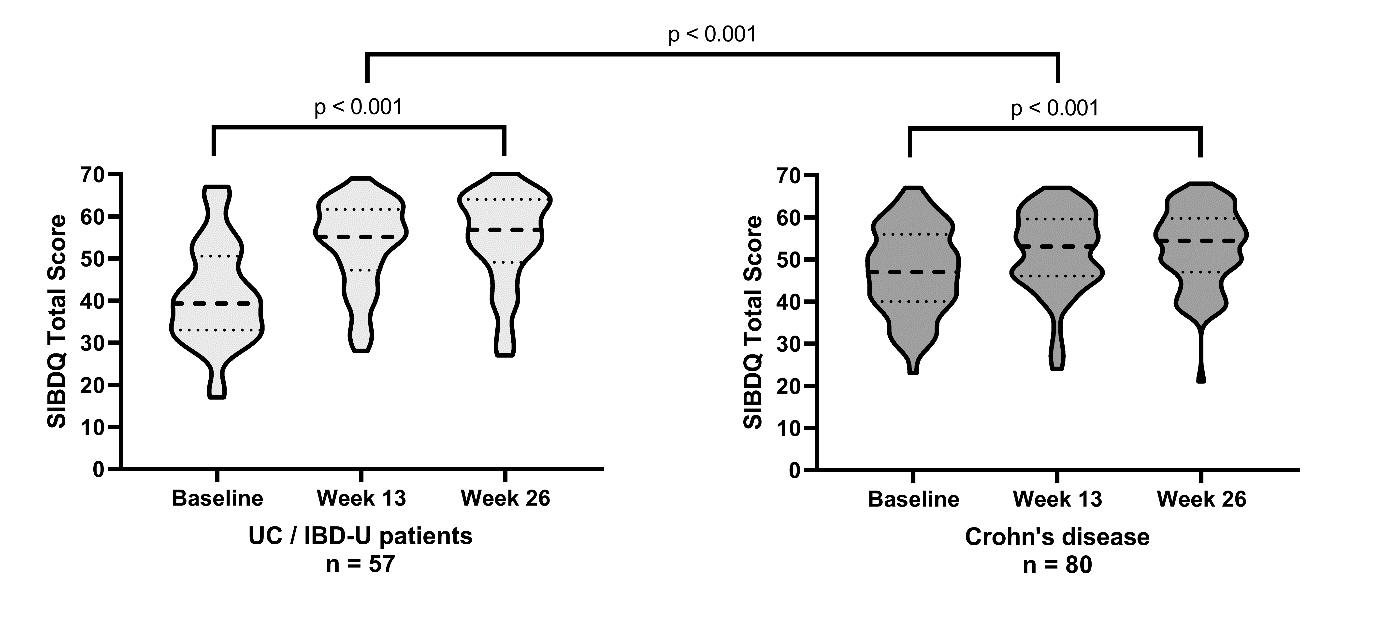


**Supplementary Figure 2.** Total short inflammatory bowel disease questionnaire (SIBDQ) scores at baseline, week 13 and week 26 stratified for Crohn’s disease and ulcerative colitis and inflammatory bowel disease unclassified patient. The thick dotted line represents the median value and the thin dotted lines represent the 25th and 75th percentiles. UC = ulcerative colitis; IBD-U = inflammatory bowel disease unclassified.

|  |  | Baseline | Week 13 | Week 26 | Improvement between week 0 - 26 | p-value |
| --- | --- | --- | --- | --- | --- | --- |
| SIBDQ Total Score | Median (IQR) | 39.3 (33.0 – 50.1) | 55.1 (47.4 – 61.3) | 56.8 (49.2 – 64.0) | 13.0 (2.8 – 23.8) | <0.001 |
| Supplementary Table 2. Short Inflammatory Bowel Disease Questionnaire total scores for ulcerative colitis and inflammatory bowel disease-unclassified patients (n=57). SIBDQ = short inflammatory bowel disease questionnaire. | | | | | | |

|  |  | Baseline | Week 13 | Week 26 | Improvement between week 0 - 26 | p-value |
| --- | --- | --- | --- | --- | --- | --- |
| SIBDQ Total Score | Median (IQR) | 47.0 (40.0 – 55.9) | 53.1 (46.2 – 59.6) | 54.4 (47.0 – 59.1) | 6.0 (0.0 – 9.9) | <0.001 |
| Supplementary Table 3. Short Inflammatory Bowel Disease Questionnaire total scores for Crohn’s disease patients (n=80). SIBDQ = short inflammatory bowel disease questionnaire. | | | | | | |

**
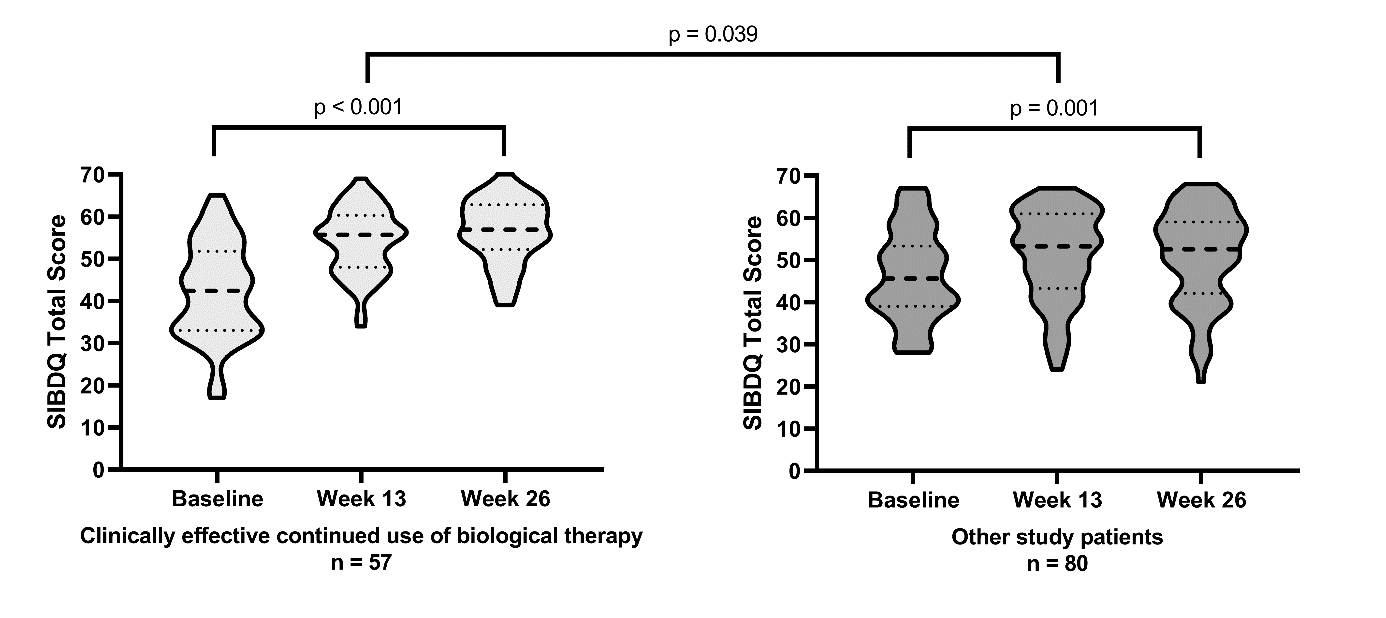
**

**Supplementary Figure 3.** Total short inflammatory bowel disease questionnaire (SIBDQ) scores at baseline, week 13 and week 26 stratified for patients with clinical disease activity at baseline (Harvey Bradshaw Index (HBI) ≥5 or Simple Clinical Colitis Activity Index (SCCAI) ≥3) and clinical response (HBI reduction ≥3 or SCCAI reduction ≥2) or clinical remission (HBI<5 or SCCAI <3) at week 26, and continued use of the of biological therapy at week 26 (n=57) compared to the remaining patients in the study population (n=80). The thick dotted line represents the median value and the thin dotted lines represent the 25th and 75th percentiles.

|  |  | Baseline | Week 13 | Week 26 | Improvement between week 0 - 26 | p-value |
| --- | --- | --- | --- | --- | --- | --- |
| SIBDQ Total Score | Median (IQR) | 42.4 (33.0 – 51.5) | 55.7 (48.0 – 60.3) | 56.9 (52.4 – 62.6) | 12.5 (5.7 – 23.0) | <0.001 |
| Supplementary Table 4. Short Inflammatory Bowel Disease Questionnaire total scores for patients with clinically effective continued of biological therapy during 26 weeks. SIBDQ = short inflammatory bowel disease questionnaire. | | | | | | |

|  |  | Baseline | Week 13 | Week 26 | Improvement between week 0 - 26 | p-value |
| --- | --- | --- | --- | --- | --- | --- |
| SIBDQ Total Score | Median (IQR) | 45.6 (39.0 – 54.9) | 53.2 (43.9 – 60.7) | 52.5 (42.1 – 59.0) | 4.6 (-0.8 – 10.6) | 0.001 |
| Supplementary Table 5. Short Inflammatory Bowel Disease Questionnaire total scores for patients with clinically effective continued of biological therapy during 26 weeks. SIBDQ = short inflammatory bowel disease questionnaire. | | | | | | |


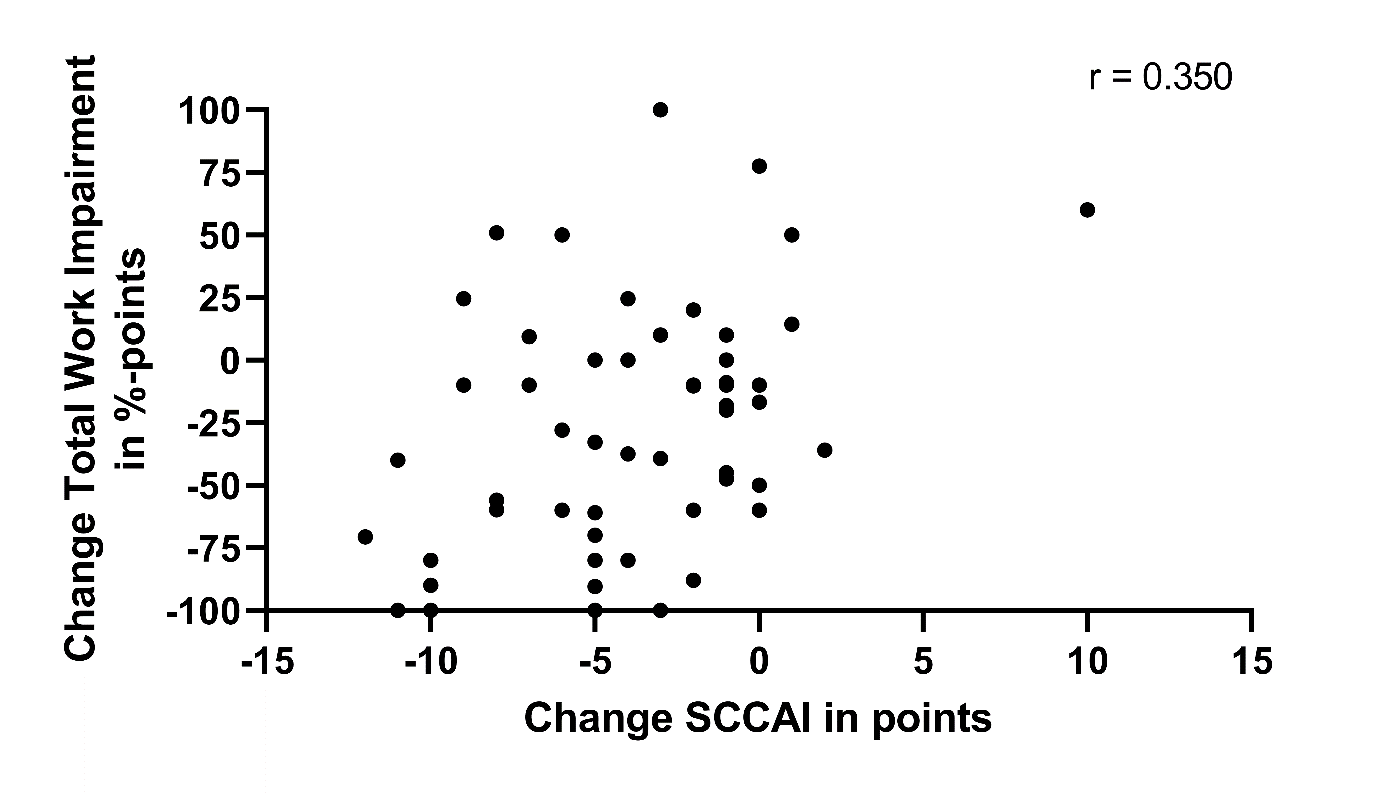


**Supplementary Figure 4.** Correlation between change in total work productivity impairment and simple clinical colitis activity index (SCCAI). Change in SCCAI scores smaller than zero indicates reduction of symptoms whereas change in SCCAI scores greater than zero indicates increase of symptoms. Change in total work impairment smaller than zero indicates decrease in work impairment whereas change in total work impairment greater than zero indicates increase in work impairment. Spearman’s rho 0.350 (n=57; p=0.010); SCCAI = simple clinical colitis activity index.


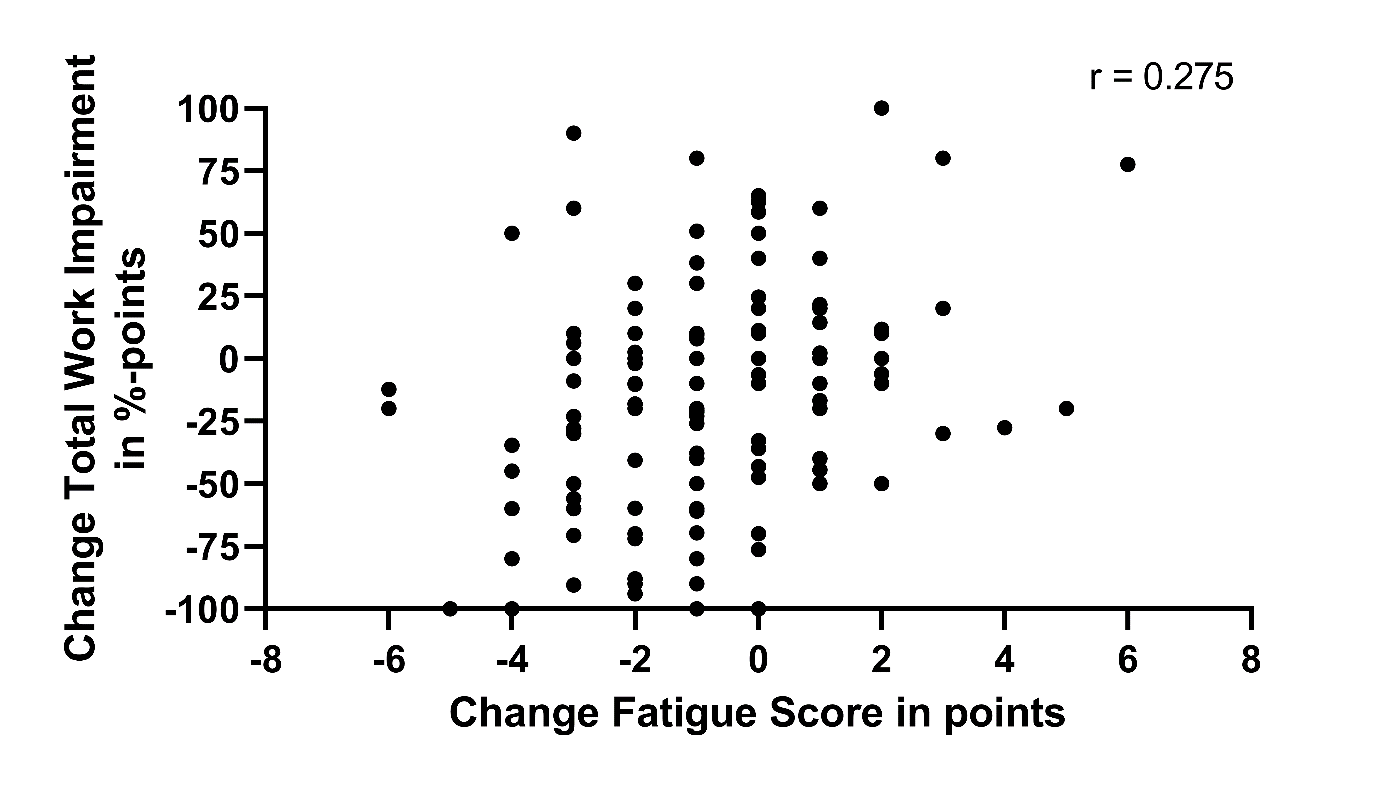


**Supplementary Figure 5.** Correlation between change in total work productivity impairment and fatigue based on question four from the Short Inflammatory Bowel Disease Questionnaire. Change smaller than zero indicates reduction of fatigue whereas change greater than zero indicates increase of fatigue. Change in total work impairment smaller than zero indicates decrease in work impairment whereas change in total work impairment greater than zero indicates increase in work impairment. Spearman’s rho 0.275 (n=137; p=0.005).


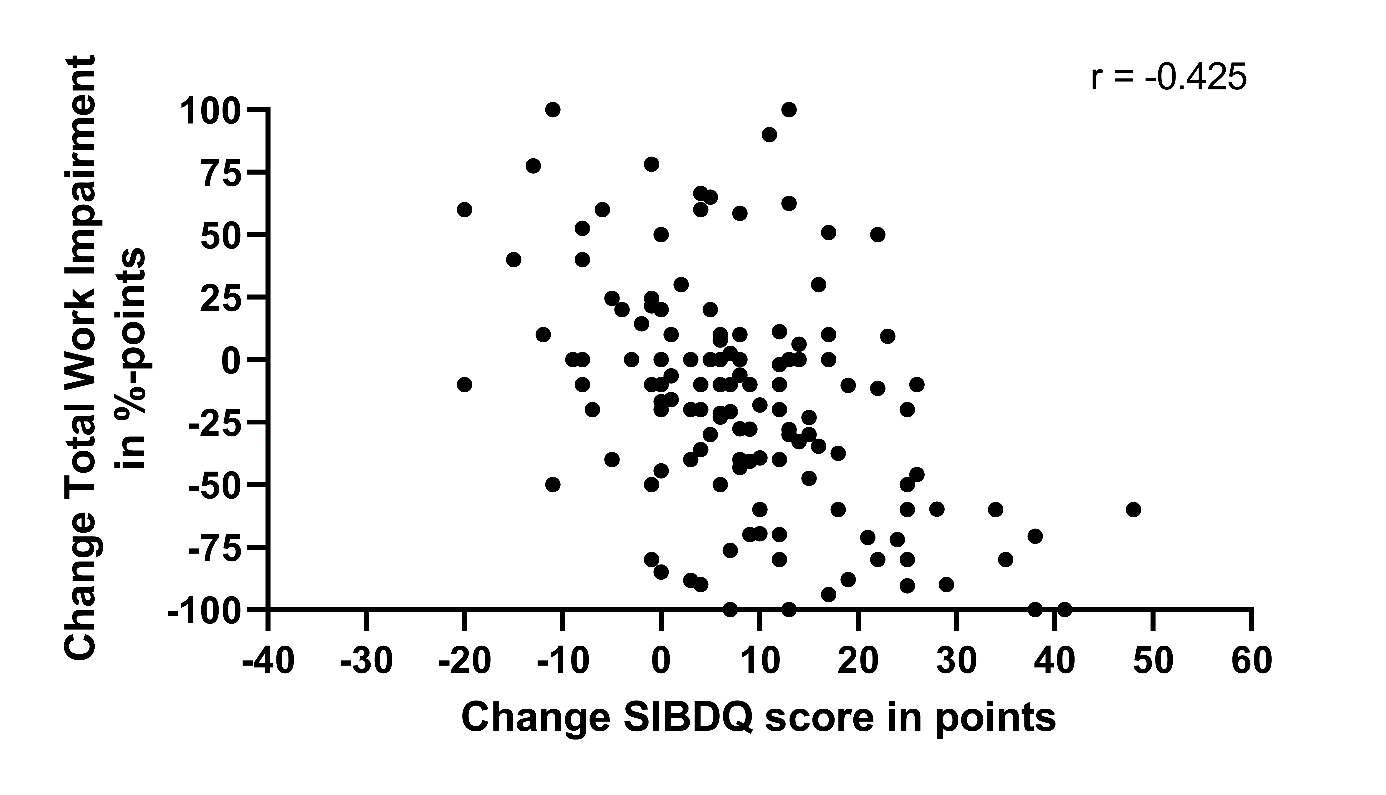


**Supplementary Figure 6.** Correlation between change in total work productivity impairment and the short Inflammatory Bowel Disease Questionnaire (SIBDQ) total score. Change smaller than zero indicates reduction of SIBDQ total score whereas change greater than zero indicates increase of SIBDQ total score. Change in total work impairment smaller than zero indicates decrease in work impairment whereas change in total work impairment greater than zero indicates increase in work impairment. Spearman’s rho -0.425 (n=137; p<0.001); SIBDQ = short inflammatory bowel disease questionnaire.
